# Interest in dietary pattern, social capital, and psychological distress: a cross-sectional study in a rural Japanese community

**DOI:** 10.1186/1471-2458-13-933

**Published:** 2013-10-07

**Authors:** Kazuyo Motohashi, Yoshihiro Kaneko, Koji Fujita, Yutaka Motohashi, Akira Nakamura

**Affiliations:** 1 Department of Public Health, Akita University Graduate School of Medicine, 1-1-1 Hondo, Akita 010-8543, Japan; 2 Department of Medical Information Science and Global Issues in Medicine, Akita University Graduate School of Medicine, 1-1-1 Hondo, Akita 010-8543, Japan

**Keywords:** Dietary pattern, Social capital, Psychological distress, Rural community, Cross-sectional study

## Abstract

**Background:**

Among life-style factors affecting mental health, dietary habits are becoming a public health concern in their relation to psychological distress and social capital. We examined associations between interest in dietary pattern, social capital, and psychological distress with a population-based cross-sectional study in rural Japan.

**Methods:**

A total of 16,996 residents of a rural town in northern Japan aged 30–79 years participated in this questionnaire survey. The questionnaire gathered data about socio-demographic variables, psychological distress, issues related to dietary habits, including interest in dietary pattern, and the social capital factors of reciprocity and sense of community belonging. Factors related to psychological distress were analyzed by using multiple logistic regression analysis.

**Results:**

A high interest in dietary pattern was significantly associated with a high level of social capital. In addition, an association between interest in dietary pattern and frequencies of intake of vegetables and fruits was confirmed. The multiple logistic regression analyses showed significant associations between interest in dietary pattern, social capital, frequency of intake of vegetables, and psychological distress after adjusting for socio-demographic variables. Low interest in dietary pattern was positively associated with psychological distress after adjusting for socio-demographic variables (OR = 2.18; 95%CI: 1.69-2.81). Low levels of both reciprocity and sense of community belonging were associated with psychological distress after adjusting for socio-demographic variables (OR = 3.46 with 95%CI of 2.10–5.71 for reciprocity, and OR = 7.42 with 95%CI of 4.64–11.87 for sense of community belonging).

**Conclusion:**

Low interest in dietary pattern, low frequency of intake of vegetables, and low levels of social capital were significantly associated with psychological distress after adjusting for socio-demographic variables.

## Background

In recent years, lifestyle factors influencing mental health have grown as a focus of concern for public health [1,2]. Alcohol abuse [3], sleep disturbance [4,5] and poor diet quality [6] have been investigated as risk factors of depression or suicide-related behaviors. Among these lifestyle factors, dietary pattern has been less intensively investigated in relation to mental health. There are several reports addressing the association of dietary pattern with depression [7,8]. The Mediterranean dietary pattern characterized by fruits, vegetables, olive oil and fish consumption has been reported to be associated with a low incidence of depression [9,10].

Regarding interest in dietary pattern or concern about healthy eating pattern, there has not been enough accumulation of data to suggest an association with psychological distress, although unhealthful dietary practices were reported to be associated with psychological distress [11]. In this study, we investigated the association of interest in dietary pattern with psychological distress in a cross-sectional study of a rural community in Japan where mental health promotion was set as a high priority on the health agenda [12]. In this study, we chose to focus on interest in dietary pattern because general loss of interest or pleasure is an important symptom of major depressive episodes [13]. Recent studies have suggested that health education on dietary patterns would be effective for the improvement of mental health [14]. Identifying a potential association between interest in dietary pattern and psychological distress has a practical implication because awareness-raising activities for increasing interest in dietary pattern would then contribute to developing an effective policy for mental health promotion in the community setting.

We hypothesized that both psychological distress and interest in dietary pattern would be influenced by the social capital factors of reciprocity and sense of community belonging, as low-levels of social capital have been reported to increase the risk of depression [15,16]. Conversely, there has been little research concerning social capital and interest in dietary pattern. Recently, Croezen et al. have reported that a low level of positive experiences of social support was associated with a low intake of fruits and vegetables [17]. As the perceived positive experience of social support was considered to be a component of social capital, their report was reflected as evidence of association between social capital and a dietary pattern consisting of vegetable and fruit intake.

**Figure 1:**
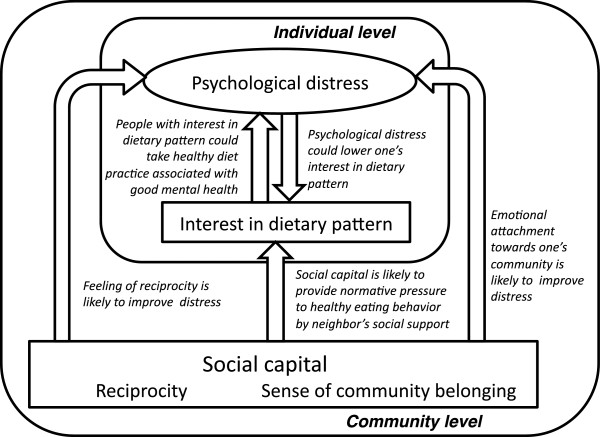
**A conceptual model is shown to explain the relationship between psychological distress, interest in dietary pattern, and social capital.** This model consists of multilevel characteristics as follows; individual level (psychological distress and interest in dietary pattern) and community level (social capital). Plausible explanations for hypothesized causal linkages (white arrows) were indicated in italics.

Figure 1 showed a conceptual model to explain the relationship between psychological distress, interest in dietary pattern and social capital. Interest in dietary pattern is likely to affect psychological distress, and *vice versa*. Both emotional attachment towards one’s community and feeling of reciprocity are likely to improve one’s feeling of distress. Furthermore, social capital could provide normative pressure to interest in dietary pattern through pathways such as neighbor’s social support. This model consists of multilevel characteristics as follows; individual level (psychological distress and interest in dietary pattern) and community level (social capital).

Thus, the objective of the present study was to assess whether psychological distress was associated with interest in dietary pattern, frequency of vegetable intake and social capital in a rural community in Japan. We conducted a survey in Akita prefecture as one of the many efforts we made to find potential clues to the high suicide rates, a reflection of high psychological distress.

## Methods

### Setting

We conducted a survey in a municipality in Akita Prefecture, which is located in northern Japan. Its population was 101,340 and the percentage of people 65 years old and over was 30.4%. The average suicide mortality rate in 2009 was 40.4 per population of 100,000, which was higher than the overall rate for Akita Prefecture for that year (38.1). Both rates were higher than the national rate of 24.4 per 100,000 population for the same year.

### Participants

The questionnaire survey was conducted between June and August 2009. The target population, aged 30–79 years old, was 16,996 residents excluding inpatients and institutional residents, such as individuals in nursing home or welfare facilities. Community volunteers or municipal employees delivered a questionnaire and received informed consent from each household and later collected the questionnaires. A total of 14,261 people (83.9%) responded.

### Questionnaire

The items examined in this study were age, sex, marital status, living arrangement (living alone vs. not alone), educational background, psychological distress, dietary habits including interest in dietary pattern, and social capital. Educational background was classified into four levels: compulsory education (9 years of schooling), high school education (12 years), junior college graduate (14 years), and university graduate or higher (16 years and over). Severity of psychological distress was assessed using a translated Japanese version of the Kessler Psychological Distress Scale (K6) [18,19]. In the Japanese K6, the Cronbach’s alpha coefficient for each measure was 0.849 [20] and its equivalence to the original English version was confirmed [21]. We defined the psychological distress group as those scoring 13 points or higher [21].

Regarding dietary habits, the questionnaires asked about interest in dietary pattern, loss of appetite, and frequency of intake of certain foods. Interest in dietary pattern was assessed by asking respondents to select one item in response to the following question: Do you have an interest in dietary pattern? Four response categories (always, often, rarely, never) were prepared. In this study, we defined “interest in dietary pattern” as “concern about healthy eating patterns”. In the surveyed municipality, health education on dietary pattern of a lower salt intake and a higher intake of fish and vegetables has been conducted against high mortality rates of cerebrovascular diseases. Thus, respondents were supposed to understand dietary pattern as the above-mentioned healthy eating pattern.

Diet history was surveyed by asking about the contents of meals the day before the surveyed day. Groups of foods were shown in the questionnaire and respondents were asked to select the relevant groups of food (multiple answers allowed). Frequencies of intake of food groups per day were counted. Groups of foods shown in the questionnaire were as follows: rice, bread, noodles, crackers, meat, fish, eggs, soybean curd and beans, vegetables, fruits, tubers and roots, milk and dairy products, cakes and confectioneries.

We measured two items specific to social capital. Questions were based on Putnam’s definition of social capital: “features of social organizations, such as networks, norms, and trust, that facilitate action and cooperation for mutual benefit” [22,23]. First, reciprocity was assessed using the following question: “Do you have a feeling that your neighbors are willing to help each other?” Second, sense of community belonging was assessed using the following question: “Do you have feelings of love for your community?” Each of the questions was answered on a four-point scale: always, often, rarely and never.

### Data analysis

The associations between interest in dietary pattern, social capital, and socio-demographic characteristics were tested using Spearman’s rank-order correlation analyses. We performed multiple logistic regression analyses to examine the associations of interest in dietary pattern and social capital with psychological distress. Among foods group, frequencies of intake of vegetables and fruits were included in the logistic regression analysis because they have been reported to be associated with depression [7,8]. First, these associations were analyzed without adjustment for other variables (model 1). Then, we analyzed the associations with adjustment for socio-demographic characteristics (age, sex, marital status, living alone, and educational background) without the variable of having an interest in diet quality (model 2). Third, we analyzed the associations with adjustment for socio-demographic characteristics without the variables of frequencies of intake of vegetables and fruits (model 3). Fourth, we analyzed the associations with adjustment for socio-demographic characteristics excluding the two social capital variables of reciprocity and sense of community belonging (model 4). Finally, we included interest in dietary pattern, frequency of intake of vegetables and social capital in the regression model and analyzed the associations with adjustment for socio-demographic characteristics (model 5).

Trends in the odds ratios for social capital as well as frequencies of food intake and socio-demographic characteristics were tested using constrained linear models [24]. All analyses were computed using SPSS 17 (Chicago, IL) statistical software.

### Ethics

This study was approved by the Ethics Committee of Akita University Graduate School of Medicine.

## Results

Of the 14,261 community residents who returned questionnaires, 11,658 (81.7%) gave complete answers for all variables included in the logistic regression analysis.

The characteristics of participants are presented in Table 1. The average age of participants was 55.6 years (SD = 13.1). In terms of educational background, high school graduates made up the largest group, comprising 54.6%. In terms of presence of any persons living together, 95.3% of participants did not live alone. The percentage of persons who had an interest in dietary pattern was 87.8%.

**Table 1:** Characteristics of participants in a surveyed town in Akita Prefecture, Japan (n = 11658)

**Variables**	**n**	**%**
Sex		
Male	5561	47.7
Female	6097	52.3
Age		
30-39 yr	1698	14.6
40-49 yr	2093	18.0
50-59 yr	3198	27.4
60-69 yr	2552	21.9
70-79 yr	2117	18.2
Marital status		
Single	1018	8.7
Married	10640	91.3
Educational background (years)		
Compulsary educationl (9 yr)	3172	27.2
High school education (12 yr)	6370	54.6
Junior colledge graduate (14 yr)	1317	11.3
University graduate and higher (more than 16 yr)	799	6.9
Living alone		
Yes	551	4.7
No	11107	95.3
Reciprocity		
Always	3521	30.2
Often	6564	56.3
Rarely	1319	11.3
Never	254	2.2
Sense of community belonging		
Always	3352	30.5
Often	6255	53.6
Rarely	1512	13.0
Never	359	3.1
Interest in dietary pattern		
Always/Often	10233	87.8
Rarely/Never	1425	12.2
Frequency of intake of vegetables		
3 times	5534	47.5
2 times	3307	28.4
1 time	1699	14.6
Never	1118	9.6
Frequency of intake of fruits		
3 times	674	5.8
2 times	1170	10.0
1 time	2468	21.2
Never	7346	63.0
Psychological distress		
Low (K6 = < 13)	11262	96.6
High (K6 > 13)	396	3.4

More than eighty per cent of the respondents felt that their neighbors were willing to help each other (30.2% selecting “always” and 56.3% selecting “often”) and the majority had a sense of community belonging (30.5% selecting “always” and 53.6% selecting “often”).

For psychological distress evaluated by the K6 scale, 3.4% participants had high psychological distress with scores greater than 13.

Table 2 shows the associations between interest in dietary pattern and two social capital items. A high interest in dietary pattern was significantly associated with a high level of reciprocity (Spearman’s ρ = 0.08, p < 0.01) and sense of community belonging (Spearman’s ρ = 0.10, p < 0.01). Those who “never” had the feeling that neighbors were willing to help each other in the community revealed the highest percentage of having no interest in dietary pattern, suggesting that the lower the level of perceived reciprocity, the higher the percentage of those who had no interest in dietary pattern (p for trend <0.01). In addition, those who never had a sense of community belonging showed the highest percentage of having no interest in dietary pattern (p for trend <0.01).

**Table 2:** Cross tabulation tables of interest in dietary pattern, social capital and frequencies of intake of vegetables and fruits

**Variables**	**Interest in dietary pattern**	
(1) Social capital	Yes	No
Reciprocity		
Always	90.7	9.3
Often	87.7	12.3
Rarely	83.9	16.1
Never	70.1	29.9
	p for trend < 0.01	
Sense of community belonging		
Always	90.9	9.1
Often	88.1	11.9
Rarely	82.8	17.2
Never	72.1	27.9
	p for trend < 0.01	
(2) Frequencies of food intake		
Intake of vegetables		
3 times	92.8	7.2
2 times	87.4	12.6
1 time	80.3	19.7
Never	75.5	24.5
	p for trend < 0.01	
Intake of fruits		
3 times	95.0	5.0
2 times	94.9	5.1
1 time	92.6	7.4
Never	84.4	15.6
	p for trend < 0.01	
(3) Demographic variables		
Sex		
Male	82.6	17.4
Female	92.5	7.5
Age		
30-39 yr	81.5	18.5
40-49 yr	84.9	15.1
50-59 yr	86.6	13.4
60-69 yr	91.2	8.8
70-79 yr	93.3	6.7
	p for trend < 0.01	
Living alone		
Alone	89.7	10.3
No	87.7	12.2
Marital status		
Single	80.9	19.1
Married	88.4	12.2
Educational background		
Compulsary educationl (9 yr)	89.9	10.2
High school education (12 yr)	86.4	13.6
Junior colledge graduate (14 yr)	89.8	10.2
University graduate and higher (more than 16 yr)	87.5	12.5
	p for trend = 0.049	

Table 2 also shows the associations between interest in dietary pattern and frequencies of intake of vegetables and fruits. The associations between interest in dietary pattern and frequencies of intake of vegetables (Spearman’s ρ = 0.23, p < 0.01) and fruits (Spearman’s ρ = 0.22, p < 0.01) were confirmed. There was a significant trend where those who had interest in dietary pattern also had higher percentages of intake of vegetables and fruits than those who had less interest in dietary pattern (p for trend <0.01).

Table 3 shows the results of multiple logistic regression analyses including interest in dietary pattern, frequency of intake of vegetables and two social capital items as explanatory variables. Frequency of intake of fruits was not shown because it was not selected as an explanatory variable in the regression model. In model 1 (without adjustment for socio-demographic variables), psychological distress was positively associated with lack of interest in dietary pattern, no intake of vegetables and lower scores on two social capital items. After adjusting for socio-demographic variables, the odds ratios of interest in dietary pattern were 2.31 (95%CI: 1.80–2.98) where frequency of intake of vegetables was excluded (model 3). After adjusting for socio-demographic variables, the odds ratios of interest in dietary pattern were 2.75 (95%CI: 2.16-3.50) where two social capital items were excluded (model 4), and 2.18 (95%CI: 1.69–2.81) where the two social capital items were included (model 5).

**Table 3:** Associations between psychological distress, interest in dietary pattern and social capital*

**Variables**	**Model 1**		**Model 2**		**Model 3**		**Model 4**		**Model 5**	
	**OR(95%CI)**	***p***	**OR(95%CI)**	***p***	**OR(95%CI)**	***p***	**OR(95%CI)**	***p***	**OR(95%CI)**	***p***
(1) Interest in dietary pattern										
Yes	Ref		-	-	Ref		Ref		Ref	
No	2.14(1.65-2.76)	<0.01	-	-	2.31(1.80-2.98)	<0.01	2.75(2.16-3.50)	<0.01	2.18(1.69-2.81)	<0.01
(2) Frequency of intake of vegetables										
None	1.81(1.28-2.55)	<0.01	2.23(1.61-3.08)	<0.01	-	-	2.20(1.60-3.03)	<0.01	1.98(1.42-2.76)	<0.01
One time	1.06(0.76-1.47)	NS	1.22(0.89-1.68)	NS	-	-	1.37(1.01-1.87)	<0.05	1.13(0.82-1.55)	NS
Two times	1.14(0.87-1.50)	NS	1.20(0.92-1.57)	NS	-	-	1.22(0.94-1.58)	NS	1.18(0.90-1.54)	NS
Three times	Ref		Ref				Ref		Ref	
(3) Social capital										
Reciprocity										
Always	Ref		Ref		Ref				Ref	
Often	1.38(0.96-1.95)	0.05	1.41(1.01-2.02)	0.05	1.39(1.00-1.93)	<0.05	-	-	1.38(1.00-1.92)	<0.05
Rarely	1.90(1.28-2.83)	<0.01	1.92(1.29-2.85)	<0.01	1.91(1.28-2.84)	<0.01	-	-	1.89(1.27-2.81)	<0.01
Never	3.51(2.13-5.78)	<0.01	3.71(2.25-6.09)	<0.01	3.46(2.10-5.71)	<0.01	-	-	3.46(2.10-5.71)	<0.01
	p for trend < 0.01		p for trednd <0.01						p for trend < 0.01	
Sense of community belonging										
Always	Ref		Ref		Ref		-	-	Ref	
Often	1.37(0.96-1.95)	0.08	1.42(1.15-2.02)	0.05	1.43(1.01-2.04)	<0.05	-	-	1.40(1.00-1.99)	<0.05
Rarely	3.71(2.51-5.51)	<0.01	4.11(2.79-6.05)	<0.01	3.99(2.71-5.87)	<0.01	-	-	3.88(2.63-5.73)	<0.01
Never	7.00(4.35-11.25)	<0.01	8.09(5.07-12.91)	<0.01	7.60(4.76-12.20)	<0.01	-	-	7.42(4.64-11.87)	<0.01
	p for trend < 0.01		p for trednd <0.01						p for trend < 0.01	

*The association between psychological distress, interest in dietary pattern, frequency of intake of vegetables, and social capital were analysed by using five logistic models.

Model 1: Without adjustment.

Model 2: Adjusted for sociodemographic characteristics of community residents (age, sex, marital status, living alone, and educational background). A variable of interest in dietary pattern was excluded in the model.

Model 3: Adjusted for sociodemographic characteristics of community residents (age, sex, marital status, living alone, and educational background). A variable of frequency of intake of vegetables was excluded in the model.

Model 4: Adjusted for sociodemographic characteristics of community residents (age, sex, marital status, living alone, and educational background). Two variables of social capital (reciprocity and sense of community belonging) were excluded in the model.

Model 5: Adjusted for sociodemographic characteristics of community residents (age, sex, marital status, living alone, and educational background). Variables of interest in dietary pattern, frequency of intake of vegetables, reciprocity and sense of community belonging were included in the model.

OR = odds ratio, CI = confidence interval.

The odds ratios of “never” having feelings that neighbors were willing to help each other (reciprocity) were 3.71 (95%CI: 2.25–6.09) where interest in dietary pattern was excluded (model 2), and 3.46 (95%CI: 2.10–5.71) where interest in dietary pattern was included (model 5). The odds ratios of “never” having feelings of love for community (sense of community belonging) were 8.09 (95%CI: 5.07–12.91) where interest in dietary pattern was excluded (model 2), and 7.42 (95%CI: 4.64–11.87) where interest in dietary pattern was included (model 5).

Finally, model 5 shows that the factors of interest in dietary pattern, frequency of intake of vegetables, reciprocity, and sense of community belonging are independently associated with psychological distress after adjusting for socio-demographic variables.

## Discussion

In this study of a Japanese rural population, both low interest in dietary pattern and low levels of the two indicators of social capital, reciprocity and sense of community belonging, were significantly associated with psychological distress after adjusting for age, gender, marital status, living alone and educational background. Furthermore, low levels of interest in dietary pattern were associated with low frequencies of intake of vegetables and fruits.

Regarding dietary patterns and mental health, Sanchez-Villegas et al. [25] reported that a Mediterranean dietary pattern decreased the incidence of depression. The Mediterranean diet is a dietary pattern characterized by a high consumption of fruits, nuts, vegetables, legumes, cereals, olive oil, and fish, a low consumption of meat and dairy products, and a moderate alcohol intake [9]. Jacka et al. [26] have reported that a traditional dietary pattern was associated with a reduced odds ratio for bipolar disorder in a population-based sample of women, where dietary patterns were classified as western, modern and traditional. Nanri et al. [27] have reported the association of a healthy Japanese dietary pattern, characterized by high intakes of vegetables, fruits, mushrooms and soy products, and reduced incidence of depressive symptoms. Furthermore, several studies have reported significant associations between dietary pattern and psychological distress [14,28].

Despite the accumulation of studies on dietary pattern and psychological distress, there are few studies on the relationship between interest in dietary pattern, which reflects a cognitive aspect of dietary behavior, and psychological distress in a population-based study. Traill et al. have reported that people who attach high importance to their own health eat a healthier diet than those who do not [29]. This meant that dietary behavior was influenced by attitudinal factors around health. In the present study, there was a tendency for people with a higher interest in dietary pattern to have higher intakes of vegetables and fruits. Healthy eating behaviors such as consuming vegetables and fruits may be driven by the attitudinal factor of interest in dietary pattern. Thus, this interest may be indirectly associated with psychological distress via healthy dietary behavior in addition to its direct association with psychological distress.

We consider the public health significance of an association between interest in dietary pattern and psychological distress to be as follows: the improvement of dietary patterns would be a favorable strategy for promoting the mental health of community residents where mental health issues such as suicide prevention are regarded as important parts of the health promotion agenda. In this context, dietary guidance for improving dietary patterns might be an effective way to intervene with community residents who have psychological distress. Health education programs aimed at elevating interest in dietary pattern could be effective countermeasures for mental health promotion and also may help lead to an improvement of health literacy on dietary habits.

Regarding social capital and psychological distress, community residents who had lower levels of psychological distress were those who had higher levels of social capital factors of reciprocity and sense of community belonging. Recently, Kim et al. reported that social capital was significantly associated with depression by a prospective study in South Korea [15]. They concluded that low level of individual-level interpersonal trust is a strong predictor of both new-onset depression (OR = 1.22) and long-term depression (OR = 1.23) after adjustment for confounders. As the measured dependent variable in the present study was psychological distress, it is not appropriate to compare our results with the report of Kim et al. However, it should be noted that the odds ratios of reciprocity as a predictor of psychological distress in our study were relatively higher (OR = 1.38–3.46) compared to the odds ratios of their report.

The present study showed that both interest in dietary pattern and social capital were independently associated with psychological distress (Model 5). In addition, there was a possibility that social capital was indirectly associated with psychological distress that was partly mediated by interest in dietary pattern.

There seem to be three possible explanations for the associations of social capital with interest in dietary pattern: facilitated access to information on healthy eating, normative pressure to adopt healthy eating behavior from neighbors’ social support, and reciprocal nonmarket exchanges of food that are characteristics of rural communities. First, people living in communities with greater levels of reciprocity could attain higher levels of health literacy by facilitated flows of health information on dietary habits through community health activities. Secondly, it is plausible that high levels of social capital in the form of reciprocity influenced interest in dietary pattern by increasing neighbors’ social support — assisting in shopping and meal preparation activities [30]. Thirdly, it has been reported that rural residents had more opportunities to receive food from neighbors than urban residents through reciprocal nonmarket food exchanges [31].

There were several limitations to this study. First, as the study design was cross-sectional, we could not discuss causal relationships. No causal relation can be suggested by this study between psychological distress and interest in dietary pattern but also that no causal relation can be suggested between social capital and psychological distress. Furthermore, it might be that people with psychological distress perceive their environment in a different way than people without psychological distress, as we measured subjectively measured social capital. Thus, it will be necessary to evaluate whether an improvement of interest in dietary pattern in community residents leads to a decreased incidence of mental distress in the community through a prospective study. Second, any differences in mental distress between the sample analyzed and the non-completing respondents may affect our results. Third, this study was conducted only in a rural community in Japan. Further study will be necessary in urban areas in order to clarify associations among the interest in dietary pattern, social capital and psychological distress.

## Conclusion

In conclusion, both low interest in dietary pattern, low frequency of intake of vegetables, and low levels of social capital factors were significantly associated with psychological distress after adjusting for socio-demographic variables.

## Competing interests

The authors declare that they have no competing interests.

## Authors’ contributions

KM, YK, YM and AN made substantial contributions to the conception and design of the study and were involved in drafting and reviewing the manuscript. KM, YK and KF contributed to the data acquisition process. KM, YK, and YM contributed to the analysis and interpretation of the data. All authors have read and approved the final manuscript.

## Pre-publication history

The pre-publication history for this paper can be accessed here:

http://www.biomedcentral.com/1471-2458/13/933/prepub

## References

[B1] Roger W Lifestyle and mental health Am Psychol 2011 66 579 592 21244124 10.1037/a0021769

[B2] Hidaka BH Depression as a disease of modernity: explanations for increasing prevalence J Affect Disord 2012 140 205 214 10.1016/j.jad.2011.12.036 22244375 PMC3330161

[B3] Han MH Kim KS Ryu SY Kang MG Park J Associations between smoking and alcohol drinking and suicidal behavior in Korean adolescents: Korea Youth Behavioral Risk Factor Surveillance, 2006 Prev Med 2009 49 248 252 10.1016/j.ypmed.2009.06.014 19573551

[B4] Baglioni C Battagliese G Feige B Spiegelhalder K Nissen C Voderholzer U Lombardo C Riemann D Insomnia as a predictor of depression: a meta-analytic evaluation of longitudinal epidemiological studies J Affect Disord 2011 135 1 19 10.1016/j.jad.2011.02.012 21300408

[B5] Krakow B Ribeiro JD Ulibarri VA Krakow J Joiner TE Jr Sleep disturbances and suicidal ideation in sleep medical center patients J Affect Disord 2011 131 422 427 10.1016/j.jad.2010.12.001 21211850

[B6] Pan X Zhang C Shi Z Soft drink and sweet food consumption and suicidal behaviours among Chinese adolescents Acta Paediatr 2011 100 e215 e222 10.1111/j.1651-2227.2011.02369.x 21627691

[B7] Oddy WH Robinson M Ambrosini GL O’Sullivan TA de Klerk NH Beilin LJ Silburn SR Zubrick SR Stanley FJ The association between dietary patterns and mental health in early adolescence Prev Med 2009 49 39 44 10.1016/j.ypmed.2009.05.009 19467256

[B8] van Kooten M de Ridder D Vollebergh W van Dorsselaer S What’s so special about eating? Examining unhealthy diet of adolescents in the context of other health-related behaviours and emotional distress Appetite 2007 48 325 332 10.1016/j.appet.2006.09.010 17095118

[B9] Drewnowski A Eichelsdoerfer P The Mediterranean diet: does it have to cost more? Public Health Nutr 2009 12 1621 1628 10.1017/S1368980009990462 19689831 PMC2849996

[B10] Sanchez-Villegas A Delgado-Rodriguez M Alonso A Schlatter J Lahortiga F Majem LS Martinez-Gonzalez MA Association of the Mediterranean dietary pattern with the incidence of depression: the Seguimiento Universidad de Navarra/University of Navarra follow-up (SUN) cohort Arch Gen Psychiatry 2009 66 1090 1098 10.1001/archgenpsychiatry.2009.129 19805699

[B11] Cohen JH Kristal AR Neumark-Sztainer D Rock CL Neuhouser ML Psychological distress is associated with unhealthful dietary practices J Am Diet Assoc 2002 102 699 703 10.1016/S0002-8223(02)90159-8 12008997

[B12] Motohashi Y Kankeo Y Sasaki H Yamaji M A decrease in suicide rates in Japanese rural towns after community-based intervention by the health promotion approach Suicide Life Threat Behav 2007 37 593 599 10.1521/suli.2007.37.5.593 17967126

[B13] American Psychiatric Association Diagnostic and statistical manual of mental disorders. Fourth edition. Text revision. DSM-IV-TR™ 2000 Washington DC: American Psychiatric Association

[B14] Lohse B Bailey RL Krall JS Wall DE Mitchell DC Diet quality is related to eating competence in cross-sectional sample of low-income females surveyed in Pennsylvania Appetite 2012 58 645 650 10.1016/j.appet.2011.11.022 22142509

[B15] Kim S-S Chung Y Perry MJ Kawachi I Subramanian SV Association between interpersonal trust, reciprocity, and depression in South Korea: a prospective analysis PLoS One 2012 7 e30602 10.1371/journal.pone.0030602 22279597 PMC3261209

[B16] Vaananen A Buunk AP Kivimaki M Vahtera J Koskenvuo M Change in reciprocity as a predictor of depressive symptoms: a prospective cohort study of Finnish women and men Soc Sci Med 2008 67 1907 1916 10.1016/j.socscimed.2008.09.015 18930340

[B17] Croezen S Picavet HSJ Haveman-Nies A Verschuren WMM de Groot LCPGM Van’t Veer P Do positive or negative experiences of social support relate to current and future health? Results from the Doetinchem Cohort Study BMC Public Health 2012 12 65 72 10.1186/1471-2458-12-65 22264236 PMC3275524

[B18] Kessler RC Andrews G Colpe LJ Hiripi E Mroczek DK Normand SL Walters EE Zaslavsky AM Short screening scales to monitor population prevalences and trends in non-specific psychological distress Psychol Med 2002 32 956 959 10.1017/s0033291702006074 12214795

[B19] Furukawa T Kessler R Andrews G Slade T The performance of the K6 and K10 screening scales for psychological distress in the Australian National Survey of Mental Health and Well-Being Psychol Med 2003 33 357 362 10.1017/S0033291702006700 12622315

[B20] Health Labour Sciences Research Grant Seijinki niokeru jisatsuyoboutaisaku no arikata nikansuru seishinhokenteki kenkyu (Mental health research on suicide prevention in adult life. My translation) 2004 Tokyo: Research report

[B21] Furukawa T Kawakami N Saitoh M Ono Y Nakane Y Nakamura Y Tachimori H Iwata N Uda H Nakane H Watanabe M Naganuma Y Hata Y Kobayashi M Miyake Y Takeshima T Kikkawa T The performance of the Japanese version of the K6 and K10 in the World Mental Health Survey Japan Int J Methods Psychiatr Res 2008 17 152 158 10.1002/mpr.257 18763695 PMC6878390

[B22] Putnam RD The prosperous community: social capital and public life Am Prospect 1993 13 35 42

[B23] Portes A Social capital: its origins and applications in modern sociology Annu Rev Sociol 1998 24 1 24 10.1146/annurev.soc.24.1.1

[B24] Selvin S Statistical analysis of epidemiologic data 1996 2 New York: Oxford University Press

[B25] Sanchez-Villegas A Verberne L Irala JD Ruiz-Canela M Toledo E Serra-Majem L Martinez-Gonzalez MA Dietary fat intake and the risk of depression: the SUN project PLoS One 2011 6 e16268 10.1371/journal.pone.0016268 21298116 PMC3027671

[B26] Jacka FN Mykletun A Berk M Bjelland I Tell GS The association between habitual diet quality and the common mental disorders in community-dwelling adults: the Hordaland Health study Psychosom Med 2011 73 483 490 10.1097/PSY.0b013e318222831a 21715296

[B27] Nanri A Kimura Y Matsushita Y Ohta M Sato M Mishima N Sasaki S Mizoue T Dietary patterns and depressive symptoms among Japanese men and women Eur J Clin Nutr 2010 64 832 839 10.1038/ejcn.2010.86 20485303

[B28] Li Y Zhang J McKeown RE Cross-sectional assessment of diet quality in individuals with a lifetime history of attempted suicide Psychiatry Res 2009 165 111 119 10.1016/j.psychres.2007.09.004 19046606

[B29] Traill WB Chambers SA Butler L Attitudinal and demographic determinants of diet quality and implications for policy targeting J Hum Nutr Diet 2012 25 87 94 10.1111/j.1365-277X.2011.01218.x 22077492

[B30] Locher JL Ritchie CS Roth DL Sawer Baker P Bodner EV Allman RM Social isolation, support, and capital and nutritional risk in an older sample: ethnic and gender differences Soc Sci Med 2005 60 747 761 10.1016/j.socscimed.2004.06.023 15571893 PMC2763304

[B31] Morton LW Bitto EA Oakland MJ Sand M Accessing food resources: rural and urban patterns of giving and getting food Agric Hum Values 2008 25 107 119

